# Use of Interleukin-12/23 Inhibitor for the Management of Acne Fulminans

**DOI:** 10.7759/cureus.50352

**Published:** 2023-12-11

**Authors:** Hannah Gier, Alexa Israeli, Austin Cusick, Dawn Merritt

**Affiliations:** 1 Dermatology, Ohio University Heritage College of Osteopathic Medicine, Dublin, USA; 2 Dermatology, Oakview Dermatology, Athens, USA; 3 Dermatology, OhioHealth Riverside Methodist Hospital, Columbus, USA

**Keywords:** il-12/23, interleukin-12/23, acne fulminans, pathophysiology of acne, accutane, ustekinumab, biologic treatment, recalcitrant acne

## Abstract

Acne fulminans (AF) is a rare disorder marked by severe eruptions of inflamed nodules, hemorrhagic crusts, and ulcers accompanied by systemic symptoms and often laboratory abnormalities. Commonly affecting adolescent males with pre-existing acne, AF has been associated with isotretinoin therapy and elevated testosterone levels. With unknown pathogenesis, lesions frequently involve the trunk and face and are managed standardly with corticosteroids and isotretinoin. Uncontrolled or recurrent cases pose challenges due to prolonged high-dose corticosteroid use with increased scarring. In this study, we present a case of AF in a 17-year-old male unresponsive to corticosteroid and isotretinoin therapy, successfully treated with ustekinumab, an interleukin (IL)-12/23 inhibitor. The introduction of ustekinumab facilitated a controlled corticosteroid taper and isotretinoin dose escalation, resulting in significant clinical improvement of skin lesions and systemic symptoms. This case report underscores the potential of ustekinumab as a viable therapeutic option in the treatment of AF, particularly in cases where corticosteroid and isotretinoin combination therapy have proven ineffective.

## Introduction

Acne fulminans (AF) is a severe and rare manifestation of inflammatory acne primarily affecting adolescent males. Characteristics of AF include the sudden onset of ulcerative nodulocystic lesions with hemorrhagic crusts, often accompanied by systemic symptoms such as fever, arthralgias, myalgias, malaise, anorexia, and hepatosplenomegaly [1-3]. Laboratory tests typically reveal anemia, leukocytosis, neutrophilia, and elevated inflammatory markers [1-3]. While the pathophysiology remains uncertain, AF is associated with a history of prolonged acne, elevated testosterone levels, and most notably, the use of isotretinoin. The standard treatment approach involves systemic corticosteroids and isotretinoin. However, refractory cases can pose significant treatment challenges and raise concerns regarding prolonged corticosteroid usage. In this case report, we introduce the successful use of the interleukin (IL)-12/23 inhibitor ustekinumab as an alternative approach for managing AF in a 17-year-old male who had shown resistance to conventional treatments.

This article was previously presented as a meeting poster at the Ohio Dermatological Annual Meeting on October 27th, 2023.

## Case presentation

**Figure 1:**
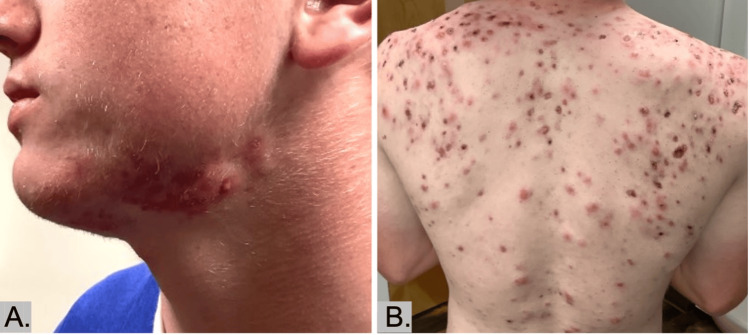
On admission. Papules, pustules, ulcerative nodules, hemorrhagic crusts, and residual scarring present on the face (A) and back (B).

A 17-year-old male presented to our clinic after months of severe, painful acne, fatigue, and malaise. Ulcerated, papulopustular acne lesions with residual scarring on his face, neck, back, and chest were visualized on the initial exam (Figure 1). Prior to presentation, the patient was treated with isotretinoin (40 mg/day) for three months, which failed to yield improvement. When restarting isotretinoin (20 mg/day), the patient’s acne again worsened. Based on history and examination at our clinic, a diagnosis of isotretinoin-induced AF with systemic symptoms was made.

At that time, a short prednisone taper was started (60 mg/day) for four days and decreased by 10 mg every four days until 20 mg was reached. With tapering, the patient experienced painful acneiform flares and symptoms consistent with corticosteroid withdrawal syndrome, including headaches, nausea, hot flashes, and malaise. Prednisone was increased again to 30 mg/day to control symptoms. To address the ongoing challenges of corticosteroid tapering and acute flares, we introduced ustekinumab as an alternative approach. Ustekinumab was initiated with a schedule of 90 mg at zero weeks, four weeks, and then every 12 weeks via subcutaneous injection, for a total of four doses, or seven months. With the use of ustekinumab, we were able to successfully taper the patient off of prednisone altogether while increasing his dose of isotretinoin with minimal to no side effects.

Over the course of seven months, ustekinumab use led to a notable reduction in nodulocystic acne and hemorrhagic ulcerations, enabling a controlled escalation of isotretinoin to 40 mg BID during ustekinumab treatment. The patient continued isotretinoin treatment until a maximum dosage of 150 mg/kg was reached, which took a total of 12 months. The prednisone taper that was started at the time of the first ustekinumab injection consisted of 30 mg/day for two weeks and then decreased to 15 mg/day for two weeks, which was then decreased by 5 mg every two weeks until 5 mg/day was reached. The patient was continued on 5 mg/day of prednisone up until month five of ustekinumab treatment. With prednisone cessation, the patient maintained little to no symptoms while completing both ustekinumab and isotretinoin therapy.

**Figure 2:**
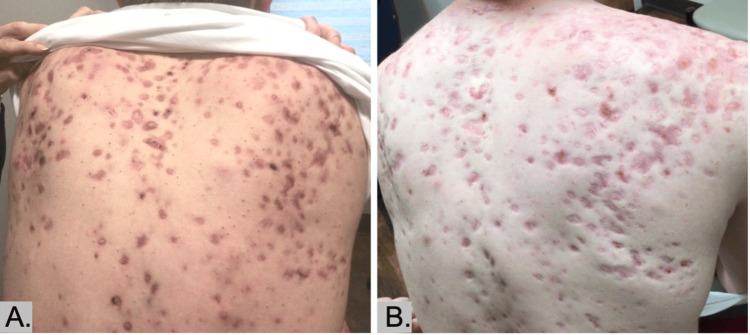
(A) Three months after initiation of ustekinumab while taking isotretinoin 30 mg/day. (B) Six months into ustekinumab treatment while on a steady dose of 40 mg/day of isotretinoin. Noting the significant reduction of nodulocystic acne.

## Discussion

AF represents a severe, eruptive form of acne with systemic symptoms such as fever, arthralgias, myalgias, malaise, hepatosplenomegaly, and rarely osteolytic lesions [4,5]. Originally referred to as “acne maligna” or “acute febrile ulcerative acne conglobata,” the term AF was introduced by Plewig and Kligman in 1975 to distinguish its unique attributes [6]. AF lesions are characterized by painful nodulocystic acne with a distinctive hemorrhagic crust, most commonly presenting on the face, back, and chest [1,3,5,7]. These severe and widespread lesions may have a detrimental effect on the self-image and confidence of affected adolescents, leading to body dysmorphic disorder and depressive syndromes in some cases [7]. The convergence of cutaneous and systemic findings associated with AF can further burden daily activities, often harming patients’ quality of life.

The demographic profile of documented AF cases predominantly comprises adolescent males between 13 and 22 years of age, often with a history of prolonged acne [6,7]. Contributing risk factors include elevated testosterone, which may result from adrenal hyperplasia or the use of anabolic steroids, and most frequently, the use of isotretinoin [1,3,6,8]. With the expanding clinical use of isotretinoin, the incidence of isotretinoin-induced AF appears to be increasing [6,9]. The condition is prominently linked to higher starting doses and the presence of comedonal acne.

While the exact etiology of AF remains unclear, its eruptive and inflammatory nature has led researchers to explore various immune mechanisms of disease. Some authors postulate that AF is triggered by a hypersensitivity reaction to *Cutibacterium acnes*, formerly known as *Propionibacterium acnes* [3,9-12]. AF is associated with the IA-1 phylotype of *Cutibacterium acnes*, which produces pro-inflammatory porphyrins, triggering an immune response that may be over-activated in those susceptible to AF [12]. In isotretinoin-induced cases, researchers suggest mechanisms of excessive granulation tissue stimulation and the release of cytokines into the dermis [6,10]. In etiologic studies of acne and inflammatory skin disease, interleukin-17A (IL-17A) is found to play a driving role [13]. When simulated by interleukin-23 (IL-23), T-helper 17 (Th17) cells enhance the production of IL-17, upregulating the inflammatory response. The IL-23/IL-17 axis is found to be active in acne lesions, with significant elevations in IL-23 and other Th17-related cytokines [13-15]. This pathway underlies several inflammatory skin conditions, including psoriasis, hidradenitis suppurativa (HS), atopic dermatitis (AD), alopecia areata, pityriasis rubra pilaris, pemphigus, and systemic sclerosis [16]. As a rare skin condition, the role of IL-23/IL-17 cytokines in AF pathogenesis remains unresearched. In effect, therapy remains untargeted.

Evidence-based recommendations for AF therapy include systemic corticosteroids combined with isotretinoin. Prednisone (0.5 mg/kg/day to 1 mg/kg/day) is commonly initiated for two to four weeks to quell cutaneous flares [6]. Once crusted ulcerations resolve, low-dose isotretinoin (0.1 mg/kg/day) is added to the regimen. Both medications are continued for a minimum of four weeks before isotretinoin is increased and corticosteroids are tapered [6]. Recommended tapering strategies involve halving the dose of corticosteroids each week until a physiologic dose is reached, followed by every-other-day dosing for two weeks. Patients who adhere to this regimen remain on corticosteroids for three to four months or longer in uncontrolled cases. If prolongation proves unsuccessful, isotretinoin may be temporarily discontinued, causing treatment delays. Alternative therapies with improved efficacy could mitigate the risks of extended therapy and enhance AF patient outcomes.

Ustekinumab is a human monoclonal antibody targeted against the p40 subunit of the proinflammatory cytokines IL-12/23 [16]. This biologic agent is approved for a variety of inflammatory conditions, including plaque psoriasis, psoriatic arthritis, Crohn’s disease, and ulcerative colitis. Off-label use of ustekinumab has demonstrated effectiveness in treating SAPHO syndrome, HS, and severe AD [2,17-20]. In patients with HS, antibodies against IL-12 and IL-23 exhibited activity against acne lesions, suggesting the potential for targeted biologics in treating more severe acne forms [13,18]. The proven efficacy of IL-12/23 inhibitors against related acneiform conditions presents an avenue for investigating their novel use in AF.

## Conclusions

Our case report illustrates the successful use of ustekinumab, an IL-12/23 inhibitor, as a novel treatment for refractory AF. By reducing reliance on prolonged corticosteroid use and enabling isotretinoin dose escalation, ustekinumab led to significant clinical improvement in our patient, with minimal to no side effects. Its known efficacy in managing various inflammatory skin conditions, such as HS, psoriasis, and severe AD, underscores its potential as a novel therapeutic option for refractory AF. Our findings support the role of ustekinumab in enhancing outcomes for AF patients who do not respond to conventional therapies.
